# Development of biomaterials for bioremediation in agriculture: the importance of correct classification of the biocompatibility of immobilized microorganisms

**DOI:** 10.1007/s10532-026-10384-w

**Published:** 2026-10-24

**Authors:** Leidaiany Pereira dos Santos, Douglas Martins de Oliveira Bessa, Fabíola Almeida Bezerra, Douglas Henrique Pereira, Grasiele Soares Cavallini, Vanessa Mara Chapla, Nelson Luis Gonçalves Dias de Souza

**Affiliations:** 1 Postgraduate Program in Chemistry, Federal University of Tocantins, Gurupi, Tocantins Brazil; 2 Postgraduate Program in Biodiversity and Biotechnology - BIONORTE, Federal University of Tocantins, Gurupi, Tocantins Brazil; 3 Department of Chemistry, Technological Institute of Aeronautics, São José dos Campos, São Paulo Brazil

**Keywords:** Pesticide, Cell, Viability, Soil, Polymer, Degradation

## Abstract

The development of biomaterials for environmental bioremediation has emerged as a sustainable and promising strategy, becoming a consolidated research area within the field of contaminated soil treatment. In this context, advances in material science and biotechnology have enabled the development of innovative immobilization supports that improve microbial stability, protection, and biodegradation performance. Among these materials, polymeric matrices combined with natural biodegradable components have demonstrated significant potential for enhancing pesticide degradation efficiency while reducing environmental impacts. However, the accurate, standardized, and robust characterization of biocompatibility remains a critical challenge, which limits the large-scale application and commercialization of these biomaterials. To address this knowledge gap, this review discusses the importance of biocompatibility classification, microbial immobilization strategies, and viability assessment methods for microorganisms entrapped in different matrices. Additionally, it underscores the imperative for the development of more dependable quality control methodologies to guarantee the efficacy, safety, and scalability of biomaterial-based bioremediation technologies.

## Introduction

The continuous growth of the human population has driven constant technological advances in agriculture (Zhang et al. 2025). One such improvement has been the use of pesticides, an approach whose implementation has intensified since the 1940s. Since their introduction, pesticide use has increased annually. According to the most recent data available, pesticide use stood at 1.80 million tons in 1990. By 2023, this figure had risen significantly to 3.73 million tons (FAO. 2025). Although pesticide use continues to rise, currently standing at 3 billion kg worldwide, only 1 percent is effective in controlling weeds. Meanwhile, the remainder applied to crops is lost through leaching or reacting with non-target plants (Tudi et al. 2021).

Pesticides include herbicides, insecticides, fungicides, nematicides, and molluscicides. Also known as xenobiotics, the best-known include insecticides, organochlorines, organophosphates, carbamate esters, pyrethroids, acetamides, triazoles, triazines, and neonicotinoids. Many of these are also classified as persistent organic pollutants (POPs) (Mateescu et al. 2024; Wang et al. 2022).

Although pesticides increase productivity, these compounds and their metabolites affect the environment, ecosystems, and biological systems (Zhang et al. 2025). This is due to their high toxicity, their capacity for bioaccumulation—owing to the absence of these chemical structures in nature —and their high lipophilicity, which results in biomagnification within the food chain (Elias et al. 2025). Other factors include their resistance to degradation by natural processes, particularly the breaking of (C–P) bonds in organophosphates, (C–Cl) bonds in organophosphates and, certainly, (C–O) bonds in ethers. In general, pesticides tend to persist in the environment and can be easily transported over long geographical distances (Elias et al. 2025; Leskovac et al. 2023; Ren et al. 2018). However, some microorganisms can degrade pesticides because their enzymatic machinery provides a catalytic advantage for cleaving bonds found in many pesticides (Efremenko et al. 2024).

In addition to organic contaminants, agricultural soils can also be affected by inorganic pollutants, such as inorganic salts, acids, and metals and metalloids. Within this group, heavy metals such as cadmium, lead, arsenic, and zinc stand out. These metals can come from different sources; in agriculture, they can result from the excessive use of inorganic salts in fertilizers and pesticides (Bonelli et al. 2017).

In this regard, it is necessary to develop sustainable alternative processes and methods by creating new materials from natural sources; for example, materials composed of microorganisms, such as fungal mycelia or free or immobilized bacterial cells, plants, or products derived from them, such as polysaccharides (Razdan & Tyagi 2027). A more standardized term for these materials is ‘biomaterials’; although this term has been widely used in regenerative medicine since 1986, its scope has expanded to include materials based on their source. Another growing demand is for biomaterials that mitigate organic and inorganic pollutants, such as pesticides and inorganic salts, that represent risks in agriculture and the environment. Over time, the field has evolved, and researchers have applied this term to non-medical materials; determining whether a material is biocompatible requires more careful assessment (Bhowmick et al. 2024; Bora et al. 2025; Razdan & Tyagi 2027).

While some scientific reviews summarize the main properties and characteristics of a biomaterial, materials with immobilized microbial cells have key physico-chemical factors, such as hydration, nutrient diffusion, and low cell density, that can determine microbial adhesion and survival. In this context, porosity provides space for biofilm formation and nutrient flow and also controls access to pesticides, oxygen, and nutrients, helping maintain metabolic activity. These factors are essential for preserving cell viability, particularly microporous cryogel substrates in bioremediation systems (De Gregorio et al. 2017; Bustos-Torrones et al. 2020; Milakin et al. 2020; Grzywaczyk et al. 2021; Kumar et al. 2025).

Some recent reviews report more comprehensive studies that classify biocompatible materials by cell viability across applications, particularly in the environmental field; however, viability is only one aspect of biocompatibility (Schmalz et al. 2017). For a biomaterial to be biocompatible, it must be characterized not only by its structure and composition, but also by data on the risks associated with its application, its performance within the system, its ecological safety for the microbiota, and its stability in soil (Schmalz et al. 2017). In the medical field, these definitions are accompanied by standardized methodologies for their assessment; however, for non-medical fields—particularly for biomaterials used in environmental bioremediation—this remains a challenge, often relying on indirect or direct assessments in terms of cell viability and biodegradability, and in many cases, these assessments are not even reported (Schmalz et al. 2017; Ismail & Mohamad 2021; Sarkar & Bhattacharjee 2025).

This narrative review critically explores how authors define biocompatibility classification standards for agricultural biomaterials, particularly for organic and inorganic bioremediation in soil and wastewater, with a focus on agricultural soil. Through a more detailed analysis, this review examines biomaterials containing immobilized microorganisms, drawing on studies of pesticide and heavy metal decontamination. This critical discussion organizes points by the biological, structural, and environmental dimensions of biocompatibility, without establishing a validated classification system.

## Biomaterials for bioremediation in agriculture: efficiency considerations for biomaterials derived from immobilized biological systems

### Biocompatibility in immobilized systems: viability and metabolic activity

Biocompatible materials can provide the physicochemical, mechanical, and biochemical properties needed to maintain the microenvironmental balance in which they are used, such as biopolymers, enzymes, or immobilized microbial cells. To maintain this balance in systems containing immobilized whole cells, a key factor to assess is cell viability, a measure that reflects the efficiency of the immobilization process (Gelmi and Schutt 2021; Efremenko et al. 2024; Ferreira et al. 2024; Gideon et al. 2024). Given that many materials are used as supports for cell adhesion via different methodologies (encapsulation, physical adsorption, covalent bonding, and cross-linking), they can maintain the conditions necessary for living cells to perform their metabolic and catalytic functions in accordance with their microenvironment (Aehle 2007; Estevinho et al. 2017; Wang et al. 2025; Wang et al. 2026).

The microenvironment of cells, particularly prokaryotic cells, favors mobility to access nutrients, maintain metabolic processes, and, at certain life-cycle stages, ensure survival by adhering to surfaces through biofilm formation. However, depending on the immobilization matrix or substrate, cellular movement required for these processes may be restricted. It may provide mechanisms that limit access to nutrients and molecules present in the chemical environment (Aehle 2007; Zamel & Khan 2021; Wang et al. 2025; Wang et al. 2026).

Another characteristic of immobilizing microbial cells is that it often maintains enzymatic metabolism, which can enhance biocatalytic activity and prolong stability. This also ensures that these materials can be easily regenerated. An important factor is that cells acting as biocatalysts must show minimal loss of catalytic activity during immobilization and reaction (Eş et al. 2015). While these properties are generally observed, other important factors include low cell flow through the immobilization support, non-compressibility, and high abrasion resistance. Unlike free cells, these factors can drastically alter the chemical environment, making further studies into the physicochemical parameters required to maintain viability necessary.

### Physico-chemical factors in microbial cell immobilization systems

Mechanical factors, such as mechanical strength, can be adjusted to improve material durability and suitability for continuous and batch flow systems (Spencer et al. 2017). Mechanical properties can provide stability, depending on the immobilization method. The authors reviewed numerous studies to demonstrate the potential and strategies of immobilization techniques, especially increased mechanical stability through crosslinking. Regarding cell mobility, porosity affects cell viability by maintaining a porous, three-dimensional network that supports nutrient diffusion and biofilm formation. Porosity also supports oxygen flow during biodegradation (Bilal et al. 2019).

Although cell immobilization can increase biomaterial viability, evaluating biosafety depends on physicochemical factors and analytical methods that determine whether a material is biosafe. Many of these tests come from the medical field and ignore the peculiarities of biomaterial microenvironments intended for non-medical use, leading to false positives. This lack of standardization and reliance on tests from other areas can limit access to concrete measures for microbiome use, increasing risks to soil microbiota when biomaterials are applied to the bioremediation of agricultural soils (Kroll et al. 2012; Zuncheddu et al. 2021).

Problems such as support turbidity and light scattering, matrix autofluorescence, adsorption or suppression of dyes and reagents, and abiotic conversion (Table 1) are common, as shown in tests with immobilized medical biomaterials, and can also occur in supports for environmental cell immobilization. Table 1 presents the consequences of interference factors and possible risk mitigation steps for the tests (Kroll et al. 2012; Zuncheddu et al. 2021; Ren & Manefield 2025).

**Table 1:** Interference factors in tests for physicochemical and biological characterization of biomaterial matrices

Interference factor	Mechanism	Consequences	Mitigation
Turbidity/Scattering	Opalescence and light scattering	False-positive dye; False-negative fluorophores	Include wash steps, adjust concentration, and use a reference wavelength.
Autoflowering	Light emission from the stand	Background noise elevation	Use of internal and external parallel controls.
Adsorption/Quenching	Dye retention or enzyme inhibition	False-negative for antioxidant and cytotoxicity tests	Substitution of incompatible assays and supplementation with serum proteins
Abiotic conversion	Cell-free catalytic oxidation	False-positive for reactive oxygen species.	Inclusion of cell-free controls and lighting control
Incomplete recovery	Cell lysis	Impossibility of cell phone reuse	Study of viability conditions

### Biodegradability

Another important factor in developing scaffolds for cell immobilization is the biodegradation rate. Scaffolds for immobilizing microorganisms in environmental applications, particularly in bioremediation, require biodegradability, including biochemical degradation factors (hydrolytic, enzymatic) and the material’s physicochemical characteristics (molecular weight, porosity/monolithicity, cross-linking density, etc.) (Mehta et al. 2025; Li et al. 2026).

## Biocompatibility beyond cell viability: methodological and conceptual limitations of the classification of biomaterials for bioremediation.

Table 2 presents a panoramic analysis of recent studies, highlighting those that used feasibility tests and classified the biomaterial as “biocompatible” directly or indirectly. It is possible to observe that there is an imprecise use of the term, with partial and insufficiently substantiated classification when the studies led to the logic of defining a biocompatible material in a single dimension, in terms of survival, viability, integrity, and cell metabolism (Moreno-Medina et al. 2014; Heidari et al. 2016).

**Table 2:** Biomaterials classified as biocompatible according to survival, growth, metabolism, or indirect definition based on specific composition: Immobilized microorganisms for application in organic and inorganic pollutant bioremediation, including agricultural research in pesticide bioremediation studies

Support	Methodology immobilization	Microorganism(s)	Application	Biocompatibility designation	Variable	Reference
Silica and Biochar	Physical adsorption	*Sphingomonas* sp. GY2B	Phenanthrene degradation	Biocompatible definition by viability	Survival	(Li et al. 2016)
Alginate microbeads with polysulfone	Prison cells and adhesion	Microbial consortium	Chlorpyrifos reduction	Viable cell count indirect definition. Because of support matrix composition	Survival	(Khalid et al. 2018)
Silkworm excrement	Physical adsorption	*Arthrobacter globiformis* D47	Degradation/Diuron	Cell density and degradation kinetics Biocompatible definition by viability	Growth and metabolism	(Liu et al. 2019)
Own pellet	-	*Cupriavidus* sp. ISTL7	Carbofuran degrading	Biocompatible definition by viability	Survival	(Gupta et al. 2019)
BC/nZVI/PSB/ PVA/AS composite	Physical adsorption	*Leclercia adecarboxylata*	Lead passivation	Pb2+ ion removal efficiency Biocompatibility by viability	Survival	(Teng et al. 2020)
(PVA/AS/CA) and 1–2% Y2 cells (PSC-Y2)	Entrapment	*Pseudomonas stutzeri* Y2	Degradation/S-triazine	Viable cell count indirect definition. because of support matrix composition	Survival	(Zhang et al. 2020)
Charcoal/ alginate	Physical adsorption	*Stenotrophomonas maltophilia* XQ08	Removal/Deltamethrin	Biodegradation kinetics/recyclability indirect because of support matrix composition	Metabolism and because of material support	(Wu et al. 2021a, b)
Different polymers	Entrapment	Consortium (*Staphylococcus capitis* CP053957.1 and *Achromobacter marplatensis* MT078618.1)	Degradation/Petroleum derivatives	Kinetics degradation Indirect definition. Because of support matrix composition	Metabolism and because of material support	(Ozyurek 2024)
Microbe-immobilized silkworm excrement (MSE)	Physical Adsorption and adhesion	*Pseudomonas aeruginosa* PS1	Phthalate esters (PAEs) degradation	Favorable biocompatibility, but there is a distinction between environmental compatibility and cytocompatibility.	Based on the survival of the cells of the biomaterial for a long period, and the term is not mentioned in the conclusions.	(Chen et al. 2026)
Vermiculite	Prisoning cells and adhesion	*Streptomyces* sp.M7	Atrazine	High biocompatibility, but there is a distinction between environmental compatibility and acceptable viability.	Based on biomaterial cell survival and the conclusions, the term is returned.	(Inorio Cappa et al. 2026)
Cellulose and alginate microfibril cryogel	Encapsulating	*Cunninghamella elegans ATCC 10028b*	Methyl Orange Dye removal	Indirect definition because of support matrix composition	Growth and metabolism	(Machado et al. 2026)

These variables are distinct and relate to cytocompatibility in terms of survival and metabolism. Integrity relates to maintaining the structure of cells adhered to the support. Thus, immobilizing microorganisms on these supports is widely used because it keeps the microenvironment stable for longer. Consequently, this increases survival and cell viability by maintaining hydration in the chemical environment and favoring cell metabolism (Alvarez et al. 2009).

Most research that classifies biomaterials as biocompatible relies on experimental evidence from cell culture, cell retention, and membrane integrity, assessed by flow cytometry using SYTO9/PI, in the degradation of phenanthrene after immobilization of *Sphingomonas* sp. GY2B in sawdust, later coated with silica in the biohybrid matrix, was classified as biocompatible even after soil-assay validation of the biomaterial. Thus, biocompatibility focuses on maintaining the viability and functionality of the immobilized microorganism and supports understanding of compatibility between the matrix and the cells, without establishing that the biomaterial is environmentally safe (Li et al. 2016).

In the formulation of *Streptomyces* sp. M7 immobilized in silkworm excrement and SE: the definition was based on the high biocompatibility with cells and colonization observed by electron microscopy, in relation to aspects of the cellular microenvironment, such as cell viability and metabolism, and to the activity of Atrazine. However, among the studies that come closest to a coherent designation, this application relates to evidence of soil biological compatibility under the experimental conditions tested, although it does not yet allow, by itself, conclusions about microbiome composition and maintenance (Inorio Cappa et al. 2026).

The favorable biocompatibility of the MSE using *Pseudomonas aeruginosa* PS1 goes beyond the survival of the immobilized microorganism in its microenvironment and suggests a distinction between environmental compatibility and cytocompatibility. However, although PAE degradation is safe for use in agricultural soil, the formulation still requires a more specific biosafety evaluation, even if favorable, after assessing the edaphic maintenance of the microbiome. Beyond biocompatibility-based evaluations, these indicators support complementary applications beyond pesticide degradation; other studies have also observed them for organic and inorganic pollutants (Teng et al. 2020; Chen et al. 2026; Machado et al. 2026). Similarly, the viability of immobilized biomaterial-based microorganisms indirectly determines chlorpyrifos and phenanthrene degradation (Li et al. 2016; Khalid et al. 2018).

In contrast, other materials such as silica-IC composites exhibit excellent reproductive stability in soil, providing a favorable microenvironment that supports both cell stability and viability. However, immobilization methods for these biomaterials mostly rely on cross-linking. This technique causes biotic stress, which can initially kill some cells. Viability or survival rate during the procedure is lower than with other methods. Nevertheless, the immobilized cells remained viable for 90–210 days after the final treatment, demonstrating prolonged storage stability (Li et al. 2020). Because the initial half-life during cross-linking is short, most immobilization methods focus on entanglement, physical adsorption, and encapsulation.

In most of the studies presented in Table 2, when the objective is to demonstrate that a material with immobilized cells is a biomaterial, only cell viability tests are performed, without the characterization of biocompatibility, pointing to the need for standardization of these terms to define the biocompatibility of a biomaterial (Gupta et al. 2019; Tang et al. 2020; Zhang et al. 2020; Li et al. 2020).

Advances in manipulating and synthesizing new biomaterials, along with biotechnological support and demand for sustainable methods, intensify this complex paradigm of nonstandardization. This broadens research beyond interactions between cells and materials. Recent research indicates a limited understanding of this expanded or restricted dimension. For example, biomaterial-based materials developed from bacterial cells immobilized in PVA-AS biochar and Silkworm (SE) droppings to degrade and reduce pollutants demonstrate that these biomaterials protect microorganisms from environmental stress and are safe for soil microbiota (Liu et al. 2019).

However, arguments against metabolite monitoring cite a lack of detailed studies on the molecule in relation to the biomaterial in silkworm (SE) droppings during diuron degradation. However, this study still characterizes the material as biocompatible. Advances in validating the system through real-field tests and in crops for 30 days show the integration of Life Cycle Assessment with in-soil stability tests (Liu et al. 2019).

In this way, standardizing biomaterial classification for bioremediation goes beyond demonstrating interactions between the cell and the material. It can also help prevent secondary pollution during biodegradation by avoiding the formation of intermediate compounds or secondary metabolites that may be more harmful than the parent compound. To confirm biocompatibility, it is necessary to evaluate environmental, ecotoxicological, and operational effects to protect the native microbiome (Liu et al. 2024; Liu et al. 2025; Liu et al. 2026). This includes standardizing measurement techniques and protocols.

Table 2**:** Biomaterials classified as biocompatible according to survival, growth, metabolism, or indirect definition based on specific composition: Immobilized microorganisms for application in organic and inorganic pollutant bioremediation, including agricultural research in pesticide bioremediation studies.

## Proposal of dimensions and criteria for correct biocompatibility classification

In science, evolving questions guide decision-making. Even a concept once considered one-dimensional becomes central to analyzing multiple dimensions that provide more information. For classifying biomaterials for non-medical purposes, the approach must be more judicious, focusing on aspects suited to, for example, agricultural or environmental applications. In this context, this section aims to inspire new perspectives of classification based on: (1) Biological biocompatibility; (2) Environmental biocompatibility.

### Biological or structural biocompatibility: Is this a new designation for non-medical biomaterials?

An intrinsic assessment of a biomaterial’s biocompatibility for medical use could use the term ‘biocompatibility’; however, for non-medical biomaterials, this term requires including the biological dimension. For agricultural and environmental applications, this need arises because terms commonly used in fields with established standards are not generally used without defining more specific criteria. ‘Biological biocompatibility’ or ‘structural biocompatibility’ is proposed as an alternative to guide the initial classification of biomaterials by characterizing the conditions for cell survival within the material, particularly when cells are immobilized on the substrate (Elias et al. 2019; Cvrček & Horáková 2019).

To assess survival, researchers applied and expanded various strategies to quantify viable cells or cell viability. According to the Organization for Economic Co-operation and Development (OECD), cell viability methods are classified as: cell damage (non-invasive cell structure), invasive cell structure damage, cell growth, and cell metabolism (Madorran et al. 2025). Chlorogenic assays, which use nitroblue tetrazolium salts (e.g., MTT), provide cell viability data based on metabolic activity to assess cytotoxic effects (Khalef et al. 2024). Furthermore, to study metabolic effects, the new Membrane Potential-Based Cell Viability Assay (MPCVA) and the DD (Direct and Dynamic) methods from Cell-Tox are experimental alternatives that may be reliable, provided their accuracy and reliability are properly investigated.

The half-maximal inhibitory concentration (IC_50_) method was used to analyze metabolism and assess cell viability in relation to cytotoxic effects, regarding the intracellular production of Ag-NP nanoparticles by the endophytic fungus *Aristolochia indica* (Kalyanasundaram et al. 2025). In 2025, the authors proposed using the CellTiter-Glo 2.0 assay to assess metabolism and non-invasive cellular damage (Sung et al. 2025). Furthermore, other metabolic assays used simple fluorescence measurements to assess the effects of stress and antifungal agents on the viability of the filamentous fungus *Magnaporthe oryzae*; these are more accessible and less costly than traditional methods (Chadha et al. 2015). The XTT assay is also a widely used metabolic measure for assessing viability; however, it has limitations in studies of Aspergillus nidulans growth and in assessing biomass (Moss et al. 2008).

Scanning imaging methods have shown promise for evaluating systems in vivo. In this context, Lavrova et al. (2021) developed a hybrid biomaterial by immobilizing yeast on an organosilica support using tetraethoxysilane and methyltriethoxysilane in the presence of a hydrophilic polymer (PEG/PVA). This demonstrated high viability in a metabolic assay. This essay assesses biocatalyst activity and examines cell integrity and morphology on the material surface using scanning electron microscopy (SEM). Quantifying microorganisms per unit surface area using this technique also enabled assessment of the effective immobilization of bacterial cells on electrospun alginate/hydrogel nanofibres (Grzywaczyk et al. 2021).

Bacterial cell viability assays generally use traditional techniques that involve measuring cell density or biomass through growth inhibition or colony-forming unit (CFU) counting. Cell density methods rely on turbidimetric measurements, making them simpler and easier to manage. However, these methods do not distinguish between dead and live bacterial cells; they merely estimate bacterial growth (Patil et al. 2014). Various interfering factors produced by metabolism or the culture medium itself affect this measurement (Khalid et al. 2018; Wacogne et al. 2024).

Viability testing has been widely accepted and used for over a century in cell counting. It remains a versatile, simple method based on culturing the inoculated microorganism in a medium in a Petri dish, followed by counting (Winding et al. 1994).

However, analyses based on bacterial cell metabolism rely on dye absorption by the membranes. A complex of proteases, lipases, and esterases hydrolyzes the dye molecules, transforming the dye’s degradation product—which is undetectable—into products that can be detected via a fluorescent signal, enabling distinction between live and dead cells (Nitsch et al. 2019). A widely used dye for determining cell viability is fluorescein diacetate (FDA); it is lipophilic and becomes more permeable to the bacterial plasma membrane’s lipid bilayer because of the molecule’s two acetate groups. After non-specific enzymatic degradation within the cell, the molecule emits a measurable signal (Nitsch et al. 2019).

The researchers determined the immobilized material’s cell viability by optimizing the protocol for immobilized bacterial cells. This method involves pre-incubating the material in a phosphate buffer at pH 7.6 at 130 rpm, then applying the protocol to measure total enzymatic activity. This is a reliable and rapid quantification protocol compared with the original method (Dzionek et al. 2022). Another conventional cell viability assay relies on the production of the fluorescent molecule resorufin, which metabolically active cells produce by converting resazurin (Calvo et al. 2025). Although this assay has limitations related to medium acidification, a method with two additional quantification steps has proven effective for estimating lactic acid bacteria viability.

The estimated proliferation of pathogenic fungi was tested using the rapid colorimetric antifungal susceptibility test with the tetrazolium salt WST-8. This method showed good agreement with traditional methods across various fungi and yeast samples (Tsukatani et al. 2014). Spore formation is common to many fungal species and is a fundamental stage in studying viability in the presence of antifungal substances and materials. The LIVE/DEAD BacLight™ kit was tested to correlate viability with spore formation in some cases (Chen and Séguin-Swartz 2002).

The advent of sophisticated genomic analysis techniques based on RNA and DNA detection has enabled the development of a methodology that uses propidium monoaide (PMA) with PCR (Jansriphibul et al. 2025). This requires understanding the processes and steps that still cause variability in cell counts, as well as the mechanisms that still need study. However, this technique is highly recommended for assessing morphological integrity, given its variability in application. GelRed also monitors bacterial viability; the dye binds strongly to DNA, allowing more accurate assessment (He et al. 2024). The study sought to minimize non-specific dye binding by adding calcium ions alongside the SYTO dye to enhance fluorescence in the E. coli O157:H7 bacterial model.

The methods presented provide important insights into viability studies and the development of analyses to characterize cellular behavior within the microenvironment. Among the most significant factors observed in analyses of microorganisms in immobilized systems, SEM imaging and fluorescent dye-based genetic methods have proven effective in assessing cell adhesion, viability, and behavior within the microenvironment with greater precision. Advanced techniques that better assess the microenvironment can predict cell behavior by monitoring specific genes, but further advances are still needed.

Therefore, when developing new materials for biological applications or those involving embedded cells, it is necessary to determine biological biocompatibility at the cell-material interface by assessing cell survival or cell viability. For example, in biomaterials for medical applications, high drug concentrations within a material can cause cytotoxicity, reducing biocompatibility. Cell viability indicates that microenvironmental conditions influence a material’s safety of use and optimal cell growth. Even if cells are not culturable, their growth can be restored later (Shukla et al. 2018).

Understanding materials’ characteristics is essential for informed application in bioremediation and agricultural contexts. One important aspect is the need to establish standardized protocols to assess the biological safety and biocompatibility of these materials, particularly for use in pesticide biodegradation (Zhang et al. 2020). However, biocompatibility analyses usually rely on the intrinsic characteristics of the material under study, and few studies standardize feasibility methods for non-medical applications. In this context, the biocompatibility assessed refers to ‘biological biocompatibility’, and one should avoid concluding that a biomaterial possesses full biocompatibility.

### Environmental biocompatibility

Environmentally safe materials for environmental and agricultural purposes are fundamental to preserving the soil ecosystem (Sawant et al. 2025). Its microbiome forms the functional basis of terrestrial ecosystems, maintaining biogeochemical cycles, fertility, and soil structure and stratification through vital transformations of nitrogen, phosphorus, and potassium. Constant exposure to xenobiotics or pesticides causes severe imbalances in microbial communities and dysbiosis in systems crucial to soil maintenance. For example, constant exposure of soil to organochlorine pesticides and endocrine disruptors has drastically reduced nitrifying bacteria in the phylum *Nitrospirae* and caused the extinction of fungi in the arbuscular mycorrhizal phylum *Glomeromycota*. These microorganisms help nutrient uptake and make nitrate and phosphorus available in the soil (Cheng et al. 2025; Egbe et al. 2021).

In this context, biomaterials designed for bioremediation can degrade xenobiotics and protect indigenous microbial communities. However, the specific effects on microbial communities need more detailed study. While they may positively affect the enrichment or maintenance of taxa essential for soil respiratory activity, they may also produce more toxic metabolites than the pesticides themselves, thereby mitigating the chemical stress caused by their addition (Wang et al. 2020; Chen et al. 2021; Wu et al. 2025).

Therefore, to classify a biomaterial for bioremediation applications, it is essential to distinguish between fundamental concepts so as not to confuse ‘biocompatible’ with ‘biodegradable’ (Espindola et al. 2026; Liu et al. 2026):

- **Physical degradation:** A process involving mechanical fragmentation, cracking, and surface deterioration.
- **Chemical degradation:** Occurs through the breaking of bonds and modifications to molecules and their functional groups due to weathering, oxidation, heating or UV radiation, and is identified using spectroscopic characterization techniques.
- **Biodegradation:** Transformation resulting from microbial metabolism itself and mediated by enzymes.
- **Complete mineralization:** the final conversion of organic compounds into inorganic compounds takes place.

Key points to bear in mind for biomaterials include the degradation of synthetic polymers and biopolymers (such as oxo-biodegradable materials, Ecoflex, and jute cellulose), which may release phytotoxic by-products or acidify the medium to below pH 4.5. For biodegradable supports such as biochar and fungal mycelium-based materials used to adsorb pollutants and heavy metals, this may remobilize harmful compounds into the environment (Miao et al. 2025; Li et al. 2026; Espindola et al. 2026; Liu et al. 2026).

Consequently, biomaterial classification cannot rely solely on immobilized cell viability to define a biomaterial as biocompatible. The assessment must include ecotoxicological tests. In Table 3, the tests presented overcome these limitations of premature classification of biomaterial biocompatibility in soil (Reyes-Cervantes et al. 2021, Chen et al. 2021, Oktaviani et al. 2024, Cao et al. 2025, Marzi et al. 2026).

**Table 3:** Microbiome evaluation Methods for determining environmental biocompatibility. Based on literature (Yu et al. 2020; Yadav et al. 2021; Zheng et al. 2022; Zmerli et al. 2023; Zhao et al. 2025)

Methods	Characteristics	WHAT they measure?
Cultivation Dependent Method	Functional to analyze viable but non-cultivable state	Abundance of bacterial and fungal fractions
Soil enzymatic activity	Immediate functional response of the heterotrophic microbiota to stress	Electron and hydrolytic transfer rate integrated into biogeochemical cycles.
Functional metabolic profile	They identify metabolites in the rhizosphere.	They analyze carbon sources by LC-MS/MS.
Molecular and omics methods	They map alpha and beta diversity without dependence on cultivation.	Quantify strategic functional genes by qPCR, 16S rRNA/ITS2 Sequencing, metagenomics, and Assembly Modeling.

### Proposed multicriteria classification

Incorporating a new criterion for classifying biomaterials is essential for their use in agriculture and the environmental sector. This matters in both academic contexts and in the practical application of methodologies aimed at transforming materials. In this context, different properties can determine at what stage or phase a new biomaterial’s development is classified (Elias et al. 2019; Sabri et al. 2023; Kiran et al. 2024).

**Fig. 1:**
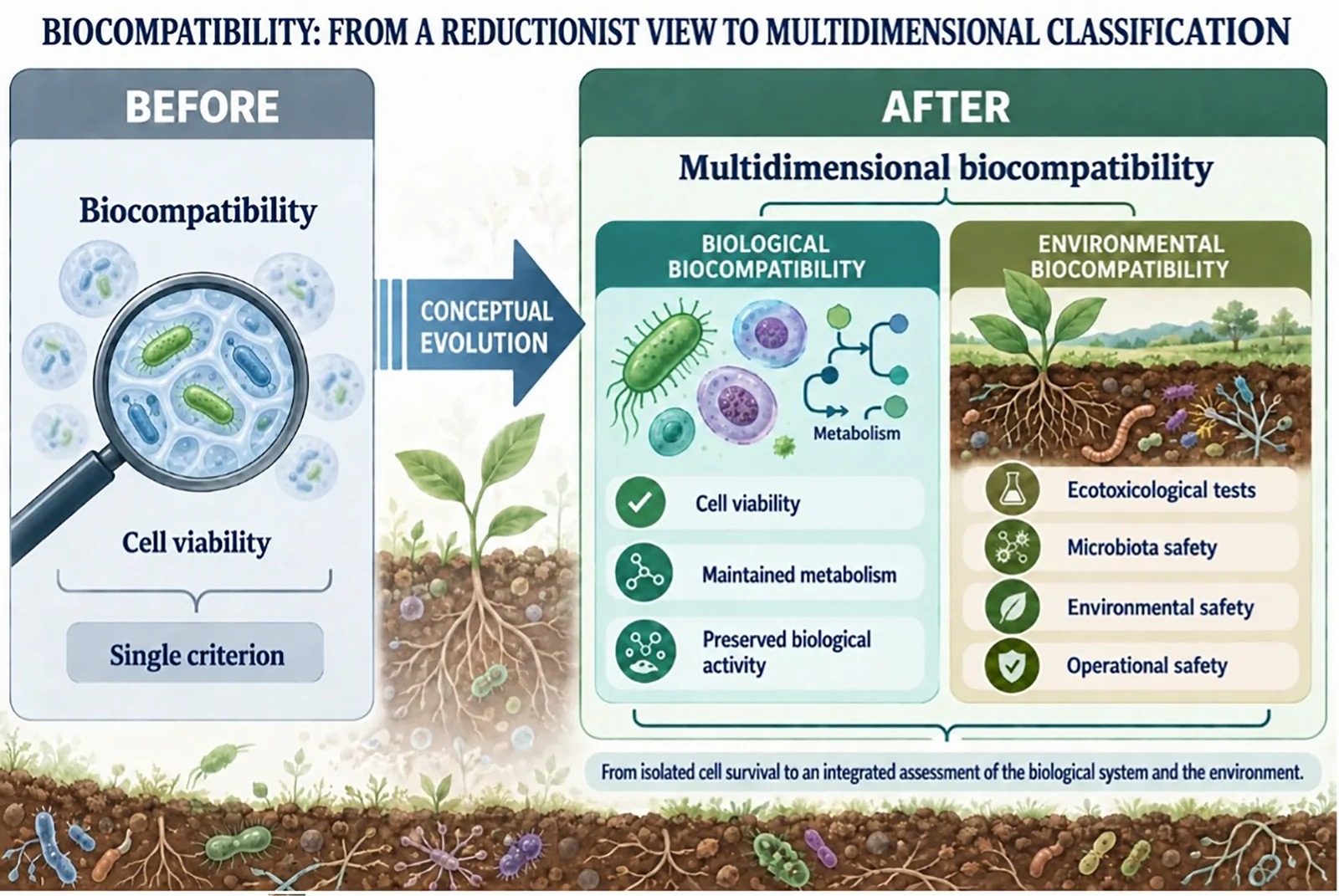
Proposed dimensional classification of biocompatibility for biomaterials applied in bioremediation

Thus, in Fig. 1, biomaterials with molecular-level characterization tests and well-defined cell-material-microenvironment interactions can be classified as biologically compatible. Materials with a broader classification level, linking safety to soil, plants, and microbiome through ecotoxicological and phytotoxicological tests, can be classified as environmentally biocompatible. Thus, this classification proposal highlights the level of consideration, and which biomaterials can evolve for future tests.

## Conclusions and perspectives

The analysis of biomaterials for agricultural and environmental applications is a highly promising field, encompassing biosafety and material efficiency and representing a crucial step toward ensuring bioproduct quality control. Developing such materials remains challenging, particularly because we need a clearer, more comprehensive understanding of their mechanisms of action, operational dynamics, and residual effects; this understanding would enable more standardized, and thus reproducible, biomaterial production. To address these challenges, many studies have used conventional, direct feasibility approaches. Nevertheless, biomaterial development specifically aimed at pesticide degradation requires further *in vitro* investigations that integrate metabolic assays with advanced surface microscopy techniques.

Developing biocompatibility protocols that directly incorporate viability assessments will ensure more reliable results and more systematic development stages for biomaterials intended for applications in soil, water, and air. This research area is inherently interdisciplinary, encompassing materials science, chemistry, biology, and molecular biology, and has the potential to transcend the limitations mentioned above.

## Data Availability

No datasets were generated or analysed during the current study.
